# Advanced Bioelectrical Signal Processing Methods: Past, Present and Future Approach—Part I: Cardiac Signals

**DOI:** 10.3390/s21155186

**Published:** 2021-07-30

**Authors:** Radek Martinek, Martina Ladrova, Michaela Sidikova, Rene Jaros, Khosrow Behbehani, Radana Kahankova, Aleksandra Kawala-Sterniuk

**Affiliations:** 1 FEECS, Department of Cybernetics and Biomedical Engineering, VSB-Technical University Ostrava, 708 00 Ostrava, Czech Republic; martina.ladrova@vsb.cz (M.L.); michaela.sidikova@vsb.cz (M.S.); rene.jaros@vsb.cz (R.J.); radana.kahankova@vsb.cz (R.K.); 2 College of Engineering, The University of Texas in Arlington, Arlington, TX 76019, USA; kb@uta.edu; 3 Faculty of Electrical Engineering, Automatic Control and Informatics, Opole University of Technology, 45-758 Opole, Poland

**Keywords:** biomedical signals, cardiac signals, electrocardiography, vectorcardiography, fetal electrocardiography

## Abstract

Advanced signal processing methods are one of the fastest developing scientific and technical areas of biomedical engineering with increasing usage in current clinical practice. This paper presents an extensive literature review of the methods for the digital signal processing of cardiac bioelectrical signals that are commonly applied in today’s clinical practice. This work covers the definition of bioelectrical signals. It also covers to the extreme extent of classical and advanced approaches to the alleviation of noise contamination such as digital adaptive and non-adaptive filtering, signal decomposition methods based on blind source separation and wavelet transform.

## 1. Introduction

Recent years have ushered in a host of important advances in biological signal sensing and microprocessor technologies. New sensors that are free from ionizing radiation, such as optical sensing devices, as well as being biocompatible for in vivo measurement, have been devised. Examples of such innovative sensors are optical-fiber based sensors and in-blood-dwelling miniature sensors [1,2]. Concurrently, multi-core microprocessors and field-programmable gate arrays (FPGA) have become available and affordable for a wide-range of applications [3]. These enabling technologies have created opportunities to develop and apply more sophisticated and effective noise decontamination methods to biological signals to obtain clinically useful information in real time. The wealth of various classical and new biological signal processing techniques has created a need to identify optimum methods for given clinical applications.

Through an extensive review of the latest methods of biological signal processing, this paper aims to provide a convenient means of considering various methods appropriate for the processing of bioelectrical signals for the clinical application at hand. Specifically, most of the popular methods for significant bioelectrical signals are summarized and the ones with most efficiency are presented in detail.

By the application of established methods of signal acquisition and processing, most degradation in bioelectrical signals that is caused by co-occurring interferences can be mitigated. This paper aims to present such proposed methods to the researchers in the field so that they can identify promising candidates for use with their applications.

### 1.1. Biological Signals—Basic Definition

For the purposes of this paper, a biological signal is defined as a signal that originates from a human body. Of course, such signals may exist as part of the homeostasis processes within an organ or be evoked by the use of an external stimulus. It is possible to group biological signals based on their physical characteristics generated naturally by stimulation. The most popular biological signals are those that are used in routine clinical practice. These are commonly as follows:

- Bioelectrical signals, which originate from the electric phenomena taking place on the membrane of cells;
- Bioacoustic signals [4,5], which entail the measurement of sounds that are generated by some organs due to fluidic or mechanical movements within the body;
- Biomechanical signals [6,7,8], which include measurement of the deflections in position, pace, acceleration, flow rates and pressures;
- Biochemical signals [9,10] providing information regarding the concentration of substances in body and their pH;
- Body temperature [11,12], which reflects the metabolic interactions within the body.

**Figure 1:**
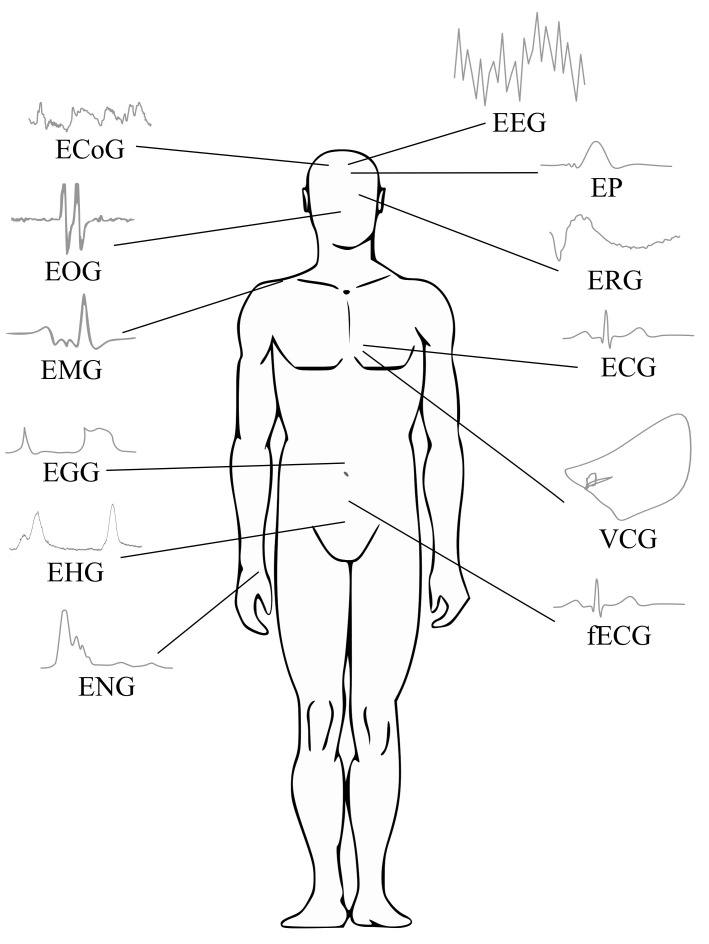
Biosignals—general scheme.

There are, of course, other types of biological signals such as bioimpedance and biomagnetic signals, which frequently are not applied due to their associated measurement complexity and limited clinical utility [13,14]. Indeed, clinical applications of the bioelectrical signals are the most prevalent. Thus, the focus of this paper is on the processing of bioelectrical signals. In Figure 1, a general scheme of various biosignals is presented.

#### Bioelectrical Signals

A bioelectrical signal is in fact the sum of action potentials of cells at an anatomical site such as the heart, brain, or skeletal muscle. The action potential is an electrical signal which accompanies a mechanical contraction of a single cell when stimulated by an electrical current, either neural or external. This electrical signal is caused by the flow of sodium (Na+), potassium (K+), chloride (Cl−), and other ions across the cell membrane [13].

The electrical activity of the cell can be divided into two phases: depolarization and repolarization. The depolarization phase represents the beginning of the action potential, with a peak value of about 20 mV for most cells. It is caused by a change of the concentration of Na+ in the cell when Na+ ions enter the cell. That is, the inside of the cell attains positive potential in relation to outside the cell. After a certain period of being in the depolarized state, the cell becomes polarized again and returns to its resting potential (ranging from −60 to −100 mV) via a process known as repolarization. During the repolarization, the predominant membrane permeability is for K+ ions, in contrast with the depolarization, when K+ ions occur considerably more slowly than Na+ ions. Since K+ concentration is much higher inside the cell than outside, there is a net efflux of K+ from the cell, which makes the inside more negative, thereby affecting repolarization back to the resting potential. The duration of the action potential for various cell types is different. For example, for the nerve and muscle cells it is about 1 ms, but for the heart muscle cells it lasts 150–300 ms [15].

Bioelectrical signals provide significant information regarding the nature of physiological activity at the single-cell level as well as at the organ level [13]. Table 1 shows the bioelectrical signal characteristics including their anatomical origin, frequency range, amplitude range and type of measurement method. In the following sections, processing methods for the bioelectrical signals listed in Table 1 are presented.

### 1.2. Noise and Interference

Each bioelectrical signal is corrupted with many types of noise and interference originating from the patient’s body or the background. These noise signals superimpose with the signal and cause its distortion in both time and frequency domains, often resulting in the total loss of the weak biological signal in the noise. Therefore, to interpret the measured signal properly and obtain diagnostic information, eliminating these interferences is necessary. The main sources of interference with the bioelectric signals (see Table 2) are as follows [41,42,43]:

- *Power line interference (PLI)* is caused by the electromagnetic field distributed through the power supply. It is the most common noise in bioelectrical signals, superposing sinusoidal harmonic components with a frequency of 50 Hz in European countries and 60 Hz in the USA and Japan.
- *Baseline wander* occurs due to breathing and changes in the electrode–skin interface, resulting in the addition of the harmonic sinusoidal component to the signal with frequencies below 1 Hz. It causes baseline instability drift and slowly changes the signal waveform. The electrode–skin interface artifacts caused by electrode movement represent a significant source of noise because of its amplitude of 200–300 mV, which represents the multiple amplitude of the measured biological signal.
- *Slight movement artifacts* can be caused by the movement of the adjacent body parts (limbs, tongue, eyes, neck, etc.). These artifacts cause a non-harmonic distortion in the signal with a frequency up to 30 Hz.
- *Myopotentials and motion artifacts* represent random broadband signals superimposed to the measured signal due to the muscle activity of the body part to which the electrode is attached or due to the patient’s bulk motion (e.g., during exercise or walking), respectively.
- *Equipment-related artifacts* are a high-frequency noise caused by the amplifiers, recording system, surrounding electromagnetic signals through power cables, or electro-surgical unit. These artifacts strongly decrease the signal’s quality and the frequency resolution and lead to the production of large-amplitude signals that can mask small features of biological signals that are important for inter alia clinical monitoring and diagnostic purposes [44,45,46]. It is also important to mention the quantization noise added by analog-to-digital signal processing hardware, or the electronic noise coupled on the biosignals due to the analog front-end hardware [47,48,49]. These artifacts result from using various data converters, where the transceiver bandwidth increases. As the data converters are usually mixed-signal components, there are issues with nonlinearity of the data. One of the most commonly modelled impairments related to the data converters is the quantization noise [50]. It occurs when a continuous random variable is converted to a discrete form or when a discrete random variable is converted to the one with fewer levels. In images or signals, the quantization noise frequently occurs during the acquisition process [51,52]. Some equipment-related noise or quality loss results from using analog-to-digital converters, which usually compress bioelectrical (in particular the ECG) signals [48].
- *Impulse noise* includes high-amplitude artifacts from digitalization and switching other electrical equipment, sharp changes of baseline due to loss of electrode–skin contact, and artifacts from electrical stimuli used when measuring the evoked activity of the investigated cells.
- *Biological signals* occurring from other structures can also be considered noise since they can overlap in time and frequency with the desired signal, see Table 1. The extraction of individual components can therefore be challenging.

**Table 2:** Classification of the common interference in bioelectrical signals.

Type of Interference		Frequency Range	Amplitude Range	Remark
Narrowband signals [41,53,54]	PLI	EU 50 Hz, USA 60 Hz	up to 5 mV	electrical wiring
	Baseline wander	up to 1 Hz 0.15–0.5 Hz	200–300 mV up to 1 mV	electrode–skin interface breathing
	Slight movements	up to 30 Hz	up to 1 mV	movements of adjacent body parts
Broadband signals [41,55]	Myopotentials and motion artifacts	20 Hz–10 kHz	up to 10 mV	muscle activity, bulk movements/exercise
	Electrosurgical noise	500 Hz–10 MHz	up to 10 mV	other medical equipment
Impulse noise [41,56]	Technical artifacts			digitalization, switching other electrical equipment
	Sharp changes of baseline			loss of electrode–skin contact
	Stimulus artifact			electrical stimuli in evoked activity measuring

## 2. Electrocardiography

Electrocardiography (ECG) is a diagnostic method, which enables the recording of the heart muscle electrical activity. The ECG is measured using the surface electrodes attached to the limbs or chest. The electrical activity of the heart, which is essential for its proper pumping function, is captured via the attached ECG electrodes. These electrical recordings provide invaluable information, ranging from the detection of arrhythmia to myocardial infarction to various cardiovascular diseases, and to disorders of the autonomic nervous system.

**Figure 2:**
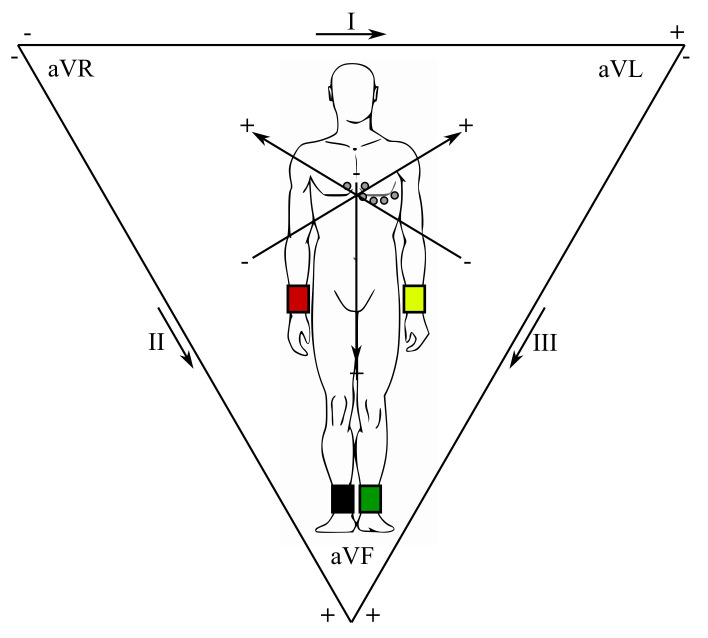
Einthoven’s triangle.

In clinical practice, a standard 12-lead ECG is obtained with 4 limb and 6 thorax electrodes (i.e., total of 10 electrodes), as certain electrodes are part of two pairs and thus provide two leads. The right foot is applied as a reference (grounding) electrode of the chest leads, formed through connection with the limb electrodes. The limb leads are usually referred to as aVR (right hand), aVL (left hand) and aVF (left foot). These three limb electrodes also make up the leads I, II, and III, known as the Einthoven’s triangle (Figure 2) [57,58]. The center of this triangle is a Wilson reference terminal. The chest leads guided towards the Wilson terminal are marked as V1–V6.

The ECG signal amplitude is within the range of 10μV to 4 mV, so it is very small and weak and therefore very prone to various disturbances or additional activity of other parts of the heart. Many of them have their own rhythms and properties in addition to the SA node and also, for example, the AV node, Purkinje fibers and ventricularity. The most important frequency of this signal is within the ranges from 0.05–100 Hz. For diagnostic purposes, the sampling frequency of 500 Hz is recommended. For long-term monitoring purposes the considered frequency range may be reduced to 0.5–50 Hz and during the stress tests up to 30–50 Hz with a lower sampling frequency of 100 Hz. Conversely, the high-resolution ECG requires a wider frequency band, which is between 0.05 and 500 Hz [15,16,44,59,60].

### 2.1. ECG Waveform

**Figure 3:**
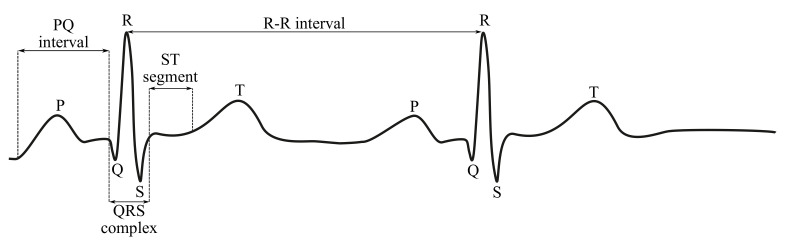
Sample ECG curve.

**Figure 4:**
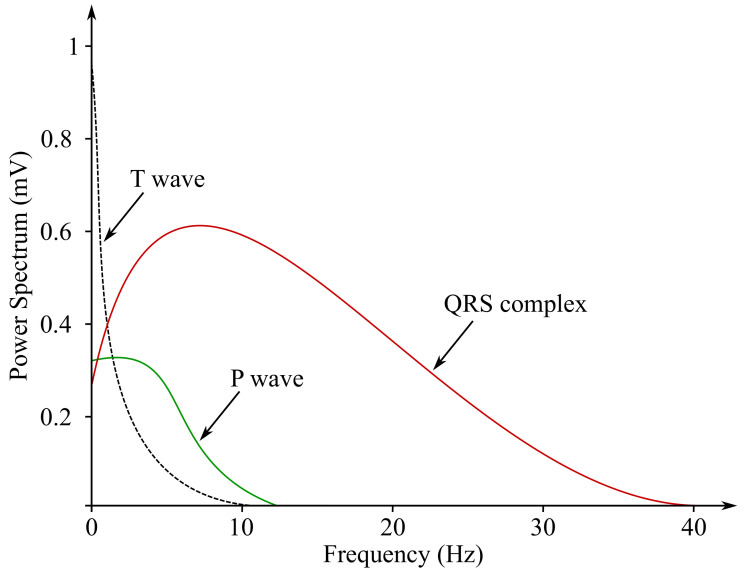
Sample ECG power spectrum.

In Figures below (Figure 3 and Figure 4), the ECG waveform is illustrated in time and frequency domains, where its parameters were marked as stated below:

- P wave is a result of the depolarization of heart atria. Due to the slow contraction of the atria and their small size, the P wave is a slow wave with a low amplitude around 0.1–0.2 mV, it lasts for approximately 60–80 ms and occurs at frequencies of up to 5 Hz with a very low power.
- PQ segment is known as an isoelectric part of the curve after the P wave, lasting about 60–80 ms. This pause helps to complete the blood transfer from the atrium to the ventricles.
- QRS complex is caused by a rapid depolarization of ventricles. It is a sharp two-staged or three-staged wave with an amplitude of 1 mV and a duration of 80–120 ms. It is the most significant part of the ECG in the frequency domain, gaining the greatest value of the spectral performance, which can be observed at the frequency range of 10–20 Hz.
- ST segment is the manifestation of the action potential plateau phase of the ventricles. It takes approximately 100–120 ms.
- T waves reflect cardiac ventricular repolarization. Their amplitude ranges within values from 0.1–0.3 mV, the duration is 120–160 ms and the frequency at which it occurs varies from 0.5 to 7 Hz [15,59,60].

### 2.2. Clinical Application

The ECG recording is usually applied for the purpose of arrhythmia detection, which means any anomaly compared with the normal sinus rhythmic activity of the heart. This may be caused by the irregular activity of the SA (sinoatrial) node, or abnormal or an additional activity of other parts of the heart. Many of them have their own rhythms and properties in addition to the SA node and also, for example, the AV node, Purkinje fibers and ventricular or atrial tissue. If the SA node is malfunctioning or its activity is limited, some of these components assume the condition of the pacemaker or can be the reason for the ectopic contractions occurring. In certain cases, it may be a different shape of the curve of the ECG, for example, a broader QRS complex, which indicates a bundle branch block or ventricular hypertrophy, increased or decreased stress level of the ST segment, turning into cardiac ischemia or myocardial infarction [61].

The other type of the ECG analysis is called heart rate variability (HRV), which means how much the heart rate (HR) changes over a finite period of observation time. The deviations in the HR can be influenced by many factors including age, gender, activity, medication taken and overall health condition. However, the HRV is mainly caused by the respiration and activity of the autonomic nervous system, which affect the heart activity. The HRV calculation is based on the R peak detection, while the RR intervals (i.e., temporal gap between R peaks of consecutive QRS complexes generated by heartbeat) are computed and the tachogram of these intervals is made for the HRV evaluation in both time and frequency domains. The results of these methods of analysis reflect the overall condition of the cardiovascular system and its neural control. The HRV analysis is frequently used during the rehabilitation process after acute myocardial infarction, chronic heart failure or after the heart transplantation, when the successive increase of the HRV is the result of convalescence or heart reinnervation. The HRV analysis is important not only for diagnosis of heart diseases, but also for detection of stroke, neuropathy related with diabetes mellitus or mental diseases. It is also an indispensable means of monitoring prenatal evolution, which helps to prevent neonatal mortality [15,17,60].

In clinical practice, the ECG recordings are conducted in several modalities including:

- **Resting ECG** is a widely used diagnostic test in clinical routines for a wide range of diseases, not necessarily of cardiac origin. The ECG is recorded for about 10 s, when the patient is at rest and in supine position. Short time limits the diagnostics of diseases of a permanent nature, which have to be investigated with the implementation of other types of measurements, such as ambulatory ECG.
- **Ambulatory monitoring** is used to identify patients with transient symptoms, for example, palpitations, lightheadedness or syncope, which can indicate arrhythmias or patients at high risk of sudden death after infarction. For 24 h or more of normal daily activities, the patient carries a solid state recording device (commonly referred to as a Holter monitor), which stores the recorded ECG.
- **Intensive care monitoring** is used for the purpose of recording and analyzing the ECG data continuously in intensive care units and coronary care units, where patients who suffered myocardial infarction or underwent heart surgery are treated. The objective of the method is to detect life-threatening arrhythmias, such as ventricular fibrillation, at an early stage. The intensive care monitoring is critically dependent on real-time signal processing: a serious event such as cardiac arrest must be detected within a few seconds so the necessary life-saving procedures can immediately start.
- **Stress test** means the ECG recording under exercise conditions when the ability of the heart to cope with physical workload is being examined. When the body works harder, the demand for oxygen increases, and the heart needs to pump more blood. Exercise usually starts at a low workload, and the load is thereafter increased progressively.
- **High-resolution ECG** enables averaging techniques for measuring the signals on the order of 1μV, which have been ignored due to their small amplitude for many years [62].

### 2.3. ECG Processing Methods

The power line interference is the most common ECG noise caused by the electromagnetic field distributed through the power supply. It adds a 50 (or 60 Hz sinusoidal harmonic component to the ECG signal.

For many years, the standard filtering methods (analogue and digital) were used in the ECG signals processing, such as the Butterworth filters, which became very popular due to their properties and simple implementation. However, with the development of the recording techniques and equipment, new methods of the ECG signal processing have to be introduced into practice, as these new diagnostics allow a more precise evaluation of the ECG parameters or faster processing during real-time monitoring [63].

For diagnostic and f-waves QRST complexes separation purposes, many methods are being applied, which can be divided into the two following categories [64]:

1. Single-lead approaches, which include among the others: the weighted average beat subtraction (WABS), the diffusion distance non-local Euclidean median filtering (DD-NLEM), and the extended Kalman smoother;
2. Multi-lead methods based on blind source separation (BSS) algorithms and those based on spatio-temporal cancellation algorithms. This approach enables the exploiting of the correlation between the channels and is usually more accurate than the single-lead-based approach.

#### 2.3.1. Digital Filtering

A Digital Filter (DF) is a system that performs mathematical operations on discrete samples of an analog signal or a discrete-time signal in order to reduce or enhance certain aspects of that signal. For the ECG processing, high-pass filter (HPF), low-pass filter (LPF), band-pass filter (BPF), and notch filter (or band-stop filter, BSF) are usually used for the elimination of the baseline wander, high-frequency noise and PLI.

Ay et al. [65] used the BPF frequency range from 0.01 to 120 Hz and a 7th order filter. The BSF was used to eliminate the 50 Hz PLI, so the implemented cut-off frequencies were from 49 to 51 Hz. For suppressing the PLI, the notch filter with the center frequency of 50 Hz can also be used [66]. Jagtap et al. [67] proposed the Butterworth filtering of the ECG, when the maximum filter order was set to 5, the filter’s stability was guaranteed. In addition, this approach was also tested in case of cascade filtering, which improved the data quality and produced better results [67,68].

#### 2.3.2. Adaptive Noise Cancellation

The Adaptive Noise Cancellation (ANC) filter consists of the filter and adaptive algorithm, which updates the filter coefficients according to the reference signal. This method is based on the principle of the interference simulation as the reference input signal, which has to be associated with this interference. The estimated noise is subtracted from the raw measured signal to obtain the filtered one. The method can be used for the purpose of eliminating the PLI, baseline wander or motion artifacts [69].

Ren et al. [44] have proposed the ANC method using two improved algorithms based on the classical LMS algorithm, normalized LMS (NLMS), and normalized block-processing LMS (BLMS). The BLMS and NLMS algorithms provide significantly better results than the classical LMS and in most cases the BLMS reaches a higher signal to noise ratio (SNR) than the NLMS. Rahman et al. [70] have proposed several simple and efficient sign-based normalized adaptive filters, which are computationally efficient due to having multiplier free-weight update loops. The proposed implementation was shown to be suitable for various applications, such as biotelemetry, where large SNRs with less computational complexity are required. These schemes mostly employ simple addition and shift operations and achieve considerable speed-up over the other LMS-based realizations. Simulation studies show that the proposed realization produces a better performance compared to existing ANC realizations in terms of the SNR and computational complexity.

In Zhang et al. [71], an adaptive filter was applied in order to significantly reduce motion artifacts in physiological signals recorded with the use of a wearable healthcare monitoring system (WHMS). The WHMS can be applied to measure the ECG, respiration and triaxial accelerometer (ACC) signals during incremental treadmill and cycle ergometry exercises. Due to the nature of these biological data and the way they were recorded it was possible to observe presence of various motion artifacts in both respiration and ECG data; therefore, in order to achieve effective and robust motion artifacts’ cancellation, three axial outputs of the ACC were applied for the purpose of the motion artifacts’ estimation by a bank of gradient adaptive Laguerre lattice (GALL) filter, and the outputs of the GALL filters were further combined with time-varying weights determined by a Kalman filter. The obtained results proved that, for the respiratory signals, the motion artifact components can be reduced, and the signal quality was efficiently improved from 0.77 to 0.96.

Tudosa et al., in [43], presented practical implementations of two derivatives of the Least Mean Square (LMS) algorithms: the Normalized LMS (NLMS) and the Sign based LMS (S-LMS). The proposed algorithms could be realized in an easy way with low computational complexity, but with a good effect on the filtering of artifacts, such as perturbations, electronic noises and power line interference signals.

#### 2.3.3. Wavelet Transform

Wavelet Transform (WT) is one of the most powerful time-frequency analysis means, which distributes continuous time signals into different scale components. After the signal reconstruction using the wavelet decomposition structure, the signal that was non-stationary and non-periodic can become a smooth signal [72,73]. The WT-based approaches are widely used due to their low complexity and high performance. They allow signal denoising with its lossless compression, R peaks detection or automated diagnosis determination of some diseases [72].

Amri et al. [74] used a discrete WT (DWT) method for the denoising of the ECG signal measured with a wireless mobile application with Android OS via Bluetooth. The signal needed to be processed in order to eliminate the noises caused by external artifacts, such as the movement of the subject or caused by the measurement electrodes. The study tested the use of *meyer*, *coiflet* and *symlet* wavelet bases—the best denoising output was achieved for the *symlet* wavelet of order 5. The WT is applied in order to remove the baseline wandering and wide-band noise using undecimated WT, wavelet *db06* and soft thresholding.

A joint approach for denoising, detection, compression and wireless transmission of the ECG signals was proposed in [75,76]. The modified biorthogonal WT (BWT) was used for the denoising, detection and lossless compression of the ECG signal. To reduce the circuit complexity, the BWT was realized using a linear phase structure. Further, it was found in this work that the usage of modified BWT increased the detection accuracy and compression ratio of the proposed design. The BWT is best suited to detecting QRS complexes. The main steps involved in the process of the ECG analysis consist of denoising and locating the different ECG peaks using adaptive slope prediction thresholding. The wireless transmission of the ECG data is very challenging due to the data conversion and power consumption.

Subramanian et al. [77] proposed compression with a dual tree complex WT (DT-CWT), which results in many wavelet coefficients with values close to zero. In order to improve the compression ratio, Set Partitioning in Hierarchical Tree (SPIHT) coding was used along with the DT-CWT for the data compression. The proposed method produced better compression ratios and reduced reconstruction errors compared to the stationary WT (SWT). Sudarshan et al. [78] used the DT-CWT method for an automated identification of the ECG signals detecting congestive heart failure from the normal ECG. In the study, they applied the DT-CWT to the ECG segments lasting 2 s and up to six levels to obtain the wavelet coefficients. The proposed method enables the accurate detection of congestive heart failure using only 2 s of the ECG signal length and hence providing sufficient time for clinicians for further investigation of the severity of the failure and treatments.

Sharma et al., in their paper (see: [79]), presented a development of a novel technique for QRS (the most distinctive feature in the ECG signal) detection. The QRS can serve as a starting point for various applications, such as, among others, the detection of other waves and segments, heart-rate calculation, derivation or respiration features. The proposed technique is based on the previously-proposed synchrosqueezed WT (SSWT), which can be obtained with the application of a post-processing technique (synchrosqueezing to the continuous WT). Following the previously-proposed SSWT, some other processing steps were applied, including the inter alia nonlinear mapping technique, which is a novel technique in the context of QRS detection applied for the purpose of R-peaks detection.

#### 2.3.4. Empirical Mode Decomposition

The Empirical Mode Decomposition (EMD) method has the properties of adaptability and signal-dependency and is suitable for biomedical signal analysis [80,81,82]. The iterative algorithm of the EMD computes maximum and minimum extremes [80,82]. The final residue signal and the Intrinsic Mode Function (IMF) are extracted after the iteration ends. In accordance with this decomposition, any signal can be represented as the sum of IMFs and the residue signal.

Suchetha et al. [45] used the EMD method for PLI removal. They applied two types of EMD methodologies: the EMD-based direct subtraction (the PLI was eliminated by directly subtracting the IMF1 from the contaminated signal) and indirect subtraction (IMF level is filtered with the BPF in order to select the band of 50 Hz interference and then the resulting signal is subtracted from the noisy ECG signal). Based on their results, the authors claim that both of the proposed methods are superior compared to typical notch filtering and partial reconstruction. As the obtained results showed, the indirect subtraction method was more efficient and accurate than the indirect method when SNR, Mean Square Error (MSE) and Root Mean Square Error (RMSE) were used as measures of algorithm performance.

#### 2.3.5. Neural Networks-Based Methods

Because the above-mentioned methods still face some challenges, such as the proper choice of the wavelet coefficient and thresholding or the accurate distinguishing of noise from the desired signal, this research also focuses on implementing neural networks and machine learning. For example, using a denoising auto-encoder (DAE) based on a deep neural network (DNN) proved to have a better performance than conventional denoising algorithms, see [83]. Deep neural networks are characterized as a multiple-layer structure, allowing more feature abstraction levels than regular networks. Based on the promising results, Chiang et al. [84] proposed DAE based on a fully convolutional network (FCN), which allows noise suppression outperforming DNN together with reducing the size of the ECG signal, which is practical for clinical diagnosis.

Antczak [85] used deep recurrent denoising neural networks that combine deep recurrent neural networks with DAE. First, the neural network is trained on artificial ECG data and is then fine-tuned with real data. This approach has shown a better performance than reference methods based on band-pass filter and wavelet transform. Deep learning models were also investigated in [86], namely Convolutional Neural Network (CNN) and the Long Short-Term Memory (LSTM) model, on both synthetic and real data. Both methods outperformed the reference wavelet-based denoising technique. Moreover, it was found that CNN achieved better results in RMS value and computational time, which was three times lower than the LSTM model.

An interesting automated detection algorithm applied for the purpose of myocardial infarction (MI) based on single-lead ECG signals was presented in [87]. The authors present two different algorithms. The detection models were built with the use of various machine learning classifiers such as k-nearest neighbor (kNN), support vector machine (SVM), ensemble bagged trees and an ensemble of the subspace of kNN.

Yang et al., in [88], presented an interesting fuzzy approach by using CNNs for the purpose of automated ECG signals’ analysis. The convolutional neural networks were used for the ECG classification without features extraction, which may result in information loss.

### 2.4. Machine Learning-Based Algorithms

In the analysis of biomedical signals, in particular ECG, various machine learning (ML) algorithms are used. Despite the fact that the classical ML methods are efficient, some novel methods have been proposed, such as the one presented by Mousavi et al. in [89], where the authors proposed a novel framework called ECG language processing (ELP) that processes the ECG signal. The ELP enables the treatment of the ECG data as a text document being analysed with the use of a natural language processing (NLP) framework. The obtained results were satisfactory and provided a new approach to the analysis and interpretation of ECG data. The proposed method was tested on single-lead and multi-lead ECG recordings from various databases.

#### 2.4.1. Clustering

The time-series clustering algorithm starts with the extraction of the features that best characterize both the shape and behavior of the analysed signal over time. It works by grouping its samples in separated clusters by means of an agglomerative clustering approach. In Rodrigues et al. [90], the authors present the results of testing the clustering method on numerous datasets in order to prove that it is independent of specific records and enables the successful detection of noisy patterns and artifacts in the analysed signals. This algorithm can be applied for the detection and noise sectioning of multiple types of noise. The proposed algorithm provides more accurate denoising results and can be successfully adapted for the classification of these signals.

#### 2.4.2. Hybrid Methods

The hybrid methods are methods that combine at least two different methods in order to use their particular advantages. In general this means that they promise a higher performance but at the expense of increased implementation complexity and computational cost.

A very interesting investigation on the effectiveness of the EMD method with a non-local mean (NLM) technique by using the value of differential standard deviation for the ECG signal’s denoising was presented in [91]. The differential standard deviation was calculated for the purpose of collecting information regarding the input noise so that the appropriate formation in the EMD and the NLM framework could be performed. The EMD framework in the proposed methodology was implemented to reduce the ECG noise. The output of the EMD framework application was passed through the NLM framework for the preservation of the edges and the cancellation of the remaining noise in the ECG signal after conducting the EMD process. The efficiency of the proposed methodology has been validated by adding white and color Gaussian noise to the clean ECG signal. The proposed denoising technique proved a lower mean of percent root mean square difference, MSE and higher mean SNR values compared to the other well-known (traditional denoising) methods at different SNR inputs.

Another efficient hybrid method for ECG denoising was proposed by Rakshit et al. in [92], which combined the EMD and Adaptive Switching Mean Filter (ASMF). The advantages of both EMD and ASMF techniques were applied for the purpose of the reduction of noises present in the ECGs signals, but with minimal distortion. Unlike the conventional EMD based techniques, which usually reject the initial IMFs or use a window-based approach to reduce the high-frequency noises, a wavelet-based soft thresholding scheme was adopted; thus, it allowed the reduction of not only the high-frequency noises but also the preservation of the QRS complexes.

Lu et al. [93] proposed, on the other hand, a novel scheme of feature selection, which applied a modified genetic algorithm using a variable-range searching strategy and the EMD. The proposed method was combined with the support vector machines (SVMs), and resulted in the development of a new ECG pattern recognition method. It worked iby first decomposing the ECG signal into the IMFs representing signal characteristics with sample oscillatory modes. Then, the modified genetic algorithm with variable-range encoding and a dynamic searching strategy was applied to optimize statistical feature subsets. Next, a statistical model based on receiver operating characteristic analysis was developed to select appropriate dominant features. Finally, the SVM-based pattern recognition model was used for the different ECG patterns’ classification. Comparative studies proved that the proposed method enables the selection of dominant features in ECG signal processing and achieves a better classification performance for lower feature dimensionality.

Another method—the improved auto-encoder based on DNN—was proposed in [94], where the WT and DNN were combined. The proposed method proved its efficiency compared to using WT and DNN methods separately. The proposed DAE reached the highest SNR values and showed a good performance in residual noise removal, which can be observed in the signal after wavelet filtering. Another combination of neural networks, in this case the feed forward back propagation neural network and WT, was proposed in [95], where the authors investigated the effectiveness of individual wavelet types. The best results were achieved by the *daubechies* wavelet (*db06*) and, in general, the method reached higher values of SNR than the particular WT approaches.

The combination of an adaptive filter based on artificial neural networks and inverse WT was described by Poungponsri et al. in [96]. The experiments presented in this paper relied on various types of interference elimination. The best SNR improvement was observed when removing PLI and the lowest, but still acceptable, one for muscle contraction artifacts’ removal. The performance in eliminating the combined noise was compared to cascaded traditional filters and separate WT—this method proved its efficiency.

In Liang et al. [97], a novel deep learning algorithm was proposed for both single- and multi-lead ECG signals’ processing. The proposed approach improves accuracy and combines the convolutional neural network (CNN) with bidirectional long short-term memory (BiLSTM).

#### 2.4.3. Summary of the ECG Signals’ Processing Methods

Table 3 shows a summary of the currently used ECG signal processing methods. For an objective comparison of these methods, they were assigned to specific classes in accordance with the following criteria:

- **Overall performance** is a parameter reflecting the robustness of the method. It is divided into three groups: *low* (enables the removal of some specific types of interference but the original signal is quite distorted); *high* (the signal is processed with the preservation of its original shape, so the detailed evaluation of all signal parameters is possible); and *medium* (the signal can be preserved when using the proper parameters for noise removal, which are difficult to choose).
- **SNR improvement** determines the efficiency of the method with regards to the reference. It is divided into three categories: *low*, *medium* and *high*.
- **Computational cost** evaluates the demands of the methods in terms of computational complexity in three categories: *low*, *medium* and *high*.
- **Real-time** is a parameter defining whether the method can be used in online mode, which is very desirable in the case of hardware devices in clinical practice and for wearable devices.
- **Implementation complexity** classifies the overall complexity in terms of the deployment in clinical practice into categories of *simple*, *medium* and *complex*, in order to evaluate the economic availability of hardware and software to all patients.

## 3. Vectorcardiography

Vectorcardiography (VCG) has received a lot of attention in the past few years. Although the information obtained during the analysis of the VCG signals has been found useful in the diagnosis process of some diseases, the measurement of this method is no longer routinely performed in clinical practice; however, it is still worth mentioning. It is a method of sensing the electrical heart activity with the use of three orthogonal leads, which correspond to the cardiac vector projections in the X, Y and Z axis directions [20,21,98,99].

This method describes the spatial development of the heart cells’ tension during its activity. The resulting electrical state of the heart is represented in the VCG as an electrical dipole. The intensity and spatial orientation of this dipole are shown as a spatial vector. The evolution of the direction and size of the vector during cardiac activity is shown in the form of loops connecting both ends of the vector. The scalar ECG can then be derived by projecting the loops into a reference system based on the Einthoven triangle in the frontal and transverse form. This relationship between the ECG and the VCG suggests that the back projection from the ECG to the VCG lacks information regarding the sagittal plane, which is directly measured in the case of the VCG signals [20,21,98,99,100,101].

### 3.1. VCG Measurement

An electric dipole is mathematically expressed as a vector, which has both magnitude and direction at each point in the time domain. In order to determine this vector, it is necessary to make three measurements in accordance with the direction of the orthogonal axes (X, Y and Z). These axes define the three basic planes (horizontal, frontal and sagittal) [20,21,98,99].

**Figure 5:**
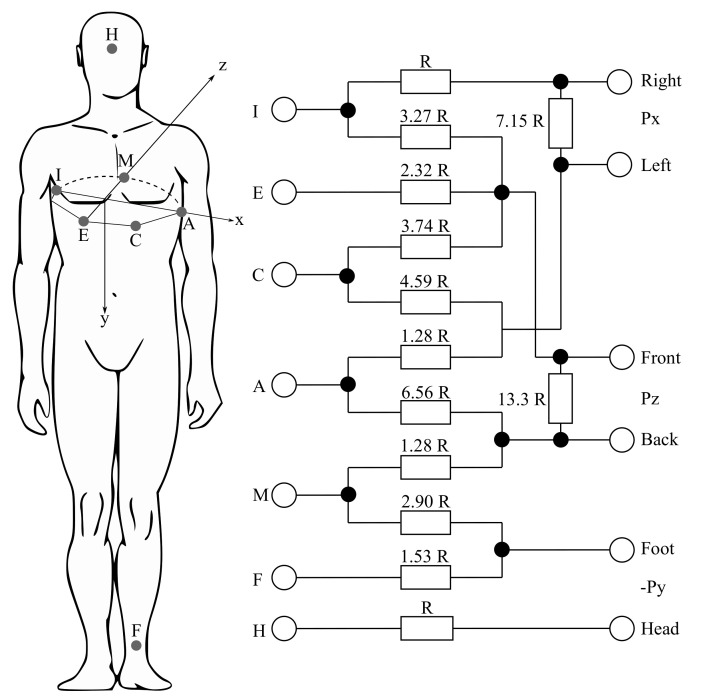
The Frank lead system [102,106].

The VCG measurements can be performed using the three following different corrected lead systems: the Frank lead system, the McFee–Parungao lead system and the SVEC III lead system. However, the Frank lead system has become the standard one for research purposes, mainly due to its simplicity and the reproducibility of the carried out measurements. The Frank lead system contains only 7 electrodes (I, E, C, A, M, F and H) and their positions are precisely defined and easily localizable for various chest shapes. However, the nature of this method implies that the Frank lead system is the most sensitive to bad electrode positioning (mostly in women and newborns). Figure 5 shows the mentioned Frank lead system. The values of the resistors are derived from the weights determined by the geometric method from the potential image of the model chest surface. By weighing the potentials from the individual electrodes, the orthogonalization is performed and the standardization of the individual leads is performed (see Equations (1)–(3)). The Frank lead system is currently the only used VCG system [21,102,103,104,105].

(1) $$\begin{array}{c} P_{X}=0.610\cdot A+0.171\cdot C-0.781\cdot I. \end{array}$$

(2) $$\begin{array}{c} P_{Y}=0.655\cdot F+0.345\cdot M-1.000\cdot H. \end{array}$$

(3) $$\begin{array}{c} P_{Z}=0.133\cdot A+0.736\cdot M-0.264\cdot I-0.374\cdot E-0.231\cdot C. \end{array}$$

The Rs from Figure 5 mean the resistors, which are connected between x- and y-components leads in order to attenuate the signals to the level of those from the z-lead [106].

### 3.2. VCG Curve

Electrical heart activity can be described with the three loops, where each represents the individual phases, where their contours, rotation or direction of the heart axis are taken into account. The three loops are listed below [20,21,98,99]:

- First and the smallest loop—the P wave;
- Second and the largest loop—the QRS complex;
- Third loop—the T wave.

**Figure 6:**
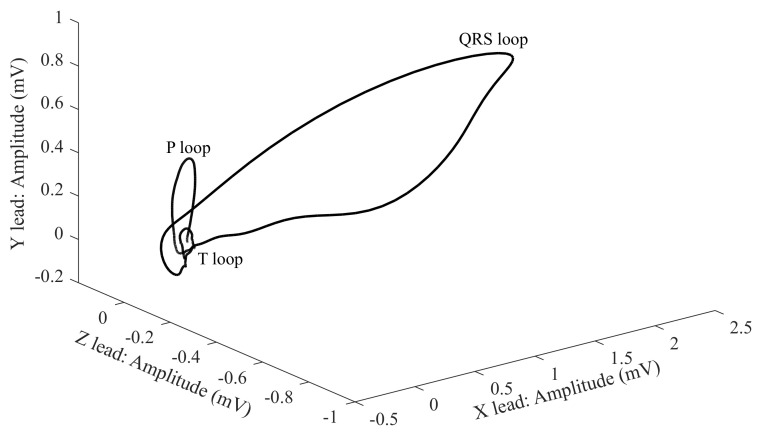
3-D image of the VCGm [102,106].

The loops can be displayed in in various views [20,21,98,99]:

1. 1-D views as three scalar records;
2. in 2-D using three planes;
3. in one 3-D view (Figure 6).

In the 1-D display, the axes are a dependence of the voltage on time. For 2-D and 3-D, the dependence between the individual leads in the mV unit is displayed. For the analysis and diagnosis of the signals, the greatest attention is paid to the QRS complex, which has an oval shape and the same direction as the heart axis [20,21,98,99].

The X-, Y- and Z-leads illustrated in Figure 6 come from the geometrical plane. This is because the VCG uses fewer leads (three leads only) than the classical ECG (12 leads), which makes the analysis less complex and improves the mobility of the measurement system [107,108].

As was mentioned previously, the VCG describes the three planes, which are perpendicular to each other. Therefore, the terms P, QRS and T are used in order to describe the spatial projection of the VCG loops on the X, Y and Z axes. By connecting these axes, the three basic planes are created [20,21,98,99]:

- The frontal plane is between the XY axes.
- The transverse plane is between the XZ axes.
- The sagittal plane is between YZ axes.

### 3.3. Clinical Applications

The advantage of the implementation of the VCG for clinical practice is its usefulness in the diagnosis of, among others, ischemic heart disease or myocardial infarction [21,109,110,111,112], as well as the other ischemic cardiac diseases. It has been shown that the combination of the ECG and the VCG provides demonstrably higher diagnostics values. Therefore, in the past, the measurements of all 15 leads (12 ECG leads and 3 VCG leads) were performed simultaneously. The current measurement with the implementation of the 15 leads is impractical for clinical application, so attention has begun to focus on the derivation of the VCG leads from the standard ECG. While this method is presently in use, there are only a few VCG workplaces that directly measure leaks [20,21,98,99]. Therefore, various transformation techniques have been developed for the purpose of synthesizing the VCG leads from the standard 12-lead ECG. The synthesized, and in some cases also directly measured, VCG leads are used for computer analysis and diagnostics. There are many transformation methods based on the coefficient transformation defined by many authors. Well-known and often used methods include, among others, the quasi-orthogonal method introduced by Kors, Bjerle and Marquette Electronics, the inverse Dower method, the Levkov transform, the Kors regression-based method and the linear regression-based methods for the P wave or the QRS complex derivation, and methods based on the artificial neural networks [21,107,108,113,114,115,116,117]. The methods based on the artificial neural networks’ implementation seems to be very efficient, because they achieve significantly higher accuracy than the standard linear transformations.

For diagnostics purposes, a number of parameters (features) have been defined, which focus on the geometric description of the loops and their mutual relations. In many cases, features derived from the VCG signals, such as the QRS-T angle, the ventricular gradient and others are used for the diagnosis of various disorders. From the morphology of the QRS loops, it is possible to describe ventricular depolarization. The most significant features are the velocity, curvature, minimal surface area, length and time of the QRS loops. These features are divided into octants for the improvement of the classification performance [20,21,98,99].

### 3.4. VCG Processing Methods

The same electrodes and almost the same configuration are used for the purpose of the VCG measurement as well as for measurement of the ECG. The VCG signals are also affected by the same noise and interferences as the ECG signals. Thus, for the processing of the VCG signals, processing methods that are similar to those for the analysis of the ECGs are used. It is very important to use suitable pre-processing of the VCG signals for the purpose of preservation and preventing corruption of the critical information that reveals the heart condition. The implementation of digital filtering methods is the most commonly used approach in VCG signal analysis. Specifically, the FIR filter and the Butterworth filter are used. In addition to the digital filters, the WT, the moving average (MA), the Kalman filter (KF), the SG filter and the principal component regression (PCR) are also applied by researchers. Table 4 shows the summary of the most popular VCG processing methods.

#### 3.4.1. Digital Filtering

In 2011, Abdelraheem [118] used the FIR filters for VCG signal processing. He wrote that the VCG signals contain both low and high frequency noise components, which need to be eliminated before any kinds of investigations take place. The baseline wandering is caused by perspiration, respiration, body movements and poor electrode contact, which have a spectral content that is usually below 1 Hz. For the processing of the VCG signals, the cascaded band-pass FIR filter with cut-off frequencies at 1 and 40 Hz was used. After filtering, the R-peak detection was successful.

In 2008, Ge [119] performed the signal pre-processing with the implementation of the low-pass filter with a cut-off frequency of 100 Hz in order to virtually eliminate the high frequency noise, the high-pass filter with a cut-off frequency of 0.4 Hz in order to suppress the residual baseline drift, and the notch filter with a cut-off frequency of 50 Hz in order to remove the power line interference.

In 2006, Guillem et al. [120] pre-processed signals with the implementation of the type II Chebyshev filter with a cut-off frequency equal to 1 Hz and with the Butterworth low-pass filter with a cut-off frequency of 50 Hz. Correa et al. in 2009 [121] used the 8th order Butterworth low-pass filter with a cut-off frequency equal to 100 Hz and the 4th order Butterworth notch filter with a cut-off frequency equal to 60 Hz for signal processing purposes. Later, in their other publications, Correa et al., in 2013 [112,122], used the 4th order Butterworth band-pass filter with cut-off frequencies at 0.2 and 100 Hz and the 2nd order Butterworth notch filter with a cut-off frequency equal to 50/60 Hz for the purpose of signal processing.

#### 3.4.2. Wavelet Transform

In 2010, Yang [123] used the DWT for VCG signal processing. In the first step, he applied a decomposed signal to the multiple wavelet filter banks with the DWT and then he quantified the cardiac recurrence dynamics for the VCG signal components within each of the wavelet scales.

Tripathy and Dandapat in 2017 [124] used the CWT and the relevance vector machine classifier for the automated grading detection of myocardial infarction from the VCG signals [125]. The results showed that the proposed method has a better performance than the other existing techniques. This approach has clinical importance due to its effective quantification of pathological changes in the VCG signal during the myocardial infarction heart ailment. They concluded that the CWT can also be used for the detection of the bundle branch block, cardiomyopathy and ventricular tachyarrhythmia pathologies from the VCG signal.

#### 3.4.3. Moving Average

Gustafson et al., in 1978 [126], removed the baseline wandering with the use of the low-pass, zero phase shift filter. The moving average filter creates a series of averages of different subsets of the full data set. The filtering is done over time, when the filter slides on a moving window with the selected number of elements and performs the averaging. They used an 800 ms window length with a sampling interval of 40 ms. They concluded that all the R-peaks in the applied records were correctly identified and that no false alarms occurred.

In order to eliminate the baseline wandering and the three-point MA filter in order to suppress the high frequency noise, in 2019, Prabhakararao and Dandapat [127] applied a Butterworth low-pass filter with a cut-off frequency equal to 0.679 Hz to each lead of the three-lead VCG signals. They claimed that the signals after this pre-processing are clinically acceptable and fed them to the feature extraction stage. They also used the three-point MA in their other publication [128].

#### 3.4.4. Savitzky-Golay Filter

Vozda in 2016 [129] compared various filters such as the FIR, the KF, the MA and the SG filters. For evaluation purposes, he chose to minimize the MSE and to maximize the correlation coefficient and to apply the analysis of the Laufberger number. Then, those individual methods were compared with each other. Based on the performed comparison of these individual methods, it follows that filtering with the use of the 2nd order SG filter with a window length of 1201 ms (with sampling frequency of 1 kHz) provided the best results and significantly outperformed the implementation of the KF, which produced the worst results.

Karsikas in 2011 [130] wrote a dissertation regarding a new method for the VCG signal processing. The noisy signals were filtered with the power line interference filter combined with the SG filter and the obtained results proved that the SG filter provided ideal signals for further processing and feature extraction.

#### 3.4.5. Principal Component Regression

Principal component regression (PCR) method was introduced by Lipponen et al. in 2010 [131] and Lipponen et al. in 2013 [132]. This method was based on modeling the QRS complex and the T wave separately. In both studies, the PCR was compared with another pre-processing method such as the previously mentioned SG filter and the DWT. Their results showed that the PCR method significantly outperformed both SG and DWT, especially in the recordings with the low SNR. They concluded that the VCG parameters, in addition to the heart rate, could lead to the better monitoring of exercise intensity and recovery.

## 4. Fetal Electrocardiography

Fetal electrocardiography (fECG) is a diagnostic method for sensing the fetal electrical heart activity. It is one of the most promising methods of electronic fetal monitoring, which provides information regarding fetal hypoxia, arrhythmia or ischemia as well as other anomalies. Most often, fetal heart rate (fHR) is the key information obtained from the fECG signal. The normal fHR is usually in the range from 110 to 150 beats per minute (bpm) [18,63,133].

Measurement of the fECG signal is performed with either invasive or non-invasive approach:

- **Invasive fECG** has several advantages. It provides a very accurate fECG signal and allows the determination of not only the fHR, but also the fHR variability, which is a parameter lost in the case of Doppler-based monitoring due to the nature of its measurement. From the measurement of the invasive fECG it is also possible to perform morphological analysis (ST segment analysis, QT analysis, etc.), which improves diagnosis of inter alia fetal hypoxia. However, measurement of the invasive fECG can be dangerous (for the fetus and also for the mother) due to the risk of infection, because it is measured with the application of transvaginal fetal scalp electrodes. This approach is also very expensive and could be used only during labor. It also requires very experienced and skilled personnel. Another disadvantage of this is that the measurement of invasive fECG is uncomfortable and it limits the movement possibilities of pregnant women. The amplitude of the fECG signal is within the range from 10μV to 3 mV [18,63,133].
- **Noninvasive fECG** is measured by means of electrodes placed on the maternal abdomen. Such signals measured on the maternal abdomen are called the abdominal ECGs (aECGs) and contain the maternal ECG, the fECG, and some kind of noise. Such noise is caused by both maternal and fetal muscle activity, respiratory activity, and so forth [18,19,63,134,135,136,137,138]. The fetal ECG signal is obtained by processing the aECG signals. The wrong configuration of electrodes’ placement could lead to an inaccurate extraction of the standardized fECG signal [18]. The non-invasive approach is an inexpensive solution and is more comfortable for pregnant women than the invasive approach. The main disadvantage of the non-invasive fECG is the low signal-to-noise ratio. There is a significant number of overlapped undesirable signals. At present, it is not possible to perform morphological analysis from the fECG signals extracted from the aECG recordings. In the near future, it can be assumed that advanced signal processing methods of the non-invasive fECG signals may soon enable morphological analysis. The amplitude of the fECG signal is in the range from 10μV to 20μV [18,63,133].

**Figure 7:**
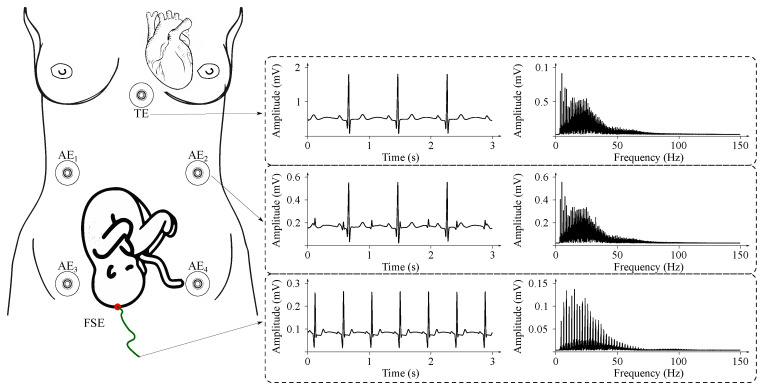
An example of signals acquired from the maternal body during non-invasive and invasive fECG monitoring in time and frequency domains: NI-fECG signals are acquired using abdominal electrodes AE1–AE5; direct invasive fECG signals are recorded by means of fetal scalp electrode (FSE); maternal ECG signal can be sensed on the maternal thorax (electrode marked as TE).

Sample fECG signals recorded during invasive and non-invasive movements are illustrated in Figure 7.

### 4.1. Fetal ECG Waveform

The shape of the fECG signal waveform is practically the same as that of the maternal ECG (mECG) signal (Figure 3), but the amplitude of the fECG signal is several times lower than that of the mECG signal. The most important frequency ranges of the fECG signals are from 0.05 to 100 Hz (the same as the mECG), but for diagnostics purposes, a sampling frequency of at least 500 Hz is recommended. For long-term monitoring purposes, the frequency range may be reduced to 0.5–50 Hz because the frequency domain of the fQRS complexes, needed for the fHR determination, lays within the range from 10 to 15 Hz. It can be noticed that the fQRS complexes overlap with the mQRS complexes, because the spectral density of mQRS and fQRS overlap within the range from 0.5 to 35 Hz [18,63,133].

### 4.2. Clinical Applications

The non-invasive fECG is one of the most promising methods for continuous fetal monitoring. This method provides unique information, which is useful for the purpose of fetal distress determination, which cannot be found with the cardiotocography (CTG). Moreover, the fECG signal carries valuable information regarding myocardial ischemia, intrapartum hypoxia or metabolic acidosis. The main advantage of the fECG compared to the CTG is that this method does not expose mother and fetus to any kind of radiation. The fECG is also more accurate for patients with a higher body mass index (BMI) [139,140]. It is a very safe and inexpensive method, which allows short term monitoring of the fHR trace compared to the CTG, which does not provide the beat-to-beat variability [18,19,63].

The possibility of morphological analysis (ST—segment analysis, QT—segment analysis, etc.) is one of the main advantages of the fECG for future clinical application, because by combining the fHR and the ST segment analysis it is possible to better diagnose hypoxia and to reduce unnecessarily performed termination of pregnancy. Although morphological analysis is currently only possible using the invasively recorded fECG data, hopefully the implementation of advanced signal processing methods could in the near future enable the performance of morphological analysis with non-invasive fECG. However, current technologies for non-invasive fECG only allow monitoring of the fHR [18,19,63].

Although morphological analysis is currently only possible using invasively recorded fECG data, it is reasonable to expect that more advanced digital processing of fECG and mECG could in the near future enable the performance of morphological analysis with non-invasive fECG.

Currently, only a few devices are commercially available for conducting non-invasive fECG monitoring, namely the Monica AN24 (2012), the Monica Novii Wireless Patch System (2014), the MERIDIAN M110 Fetal Monitoring System (2017) and PURE-trace (2017). These devices differ in the way they are applied on a tested body and in the number of electrodes [18]. For the purpose of more accurate fetal monitoring, the innovative and invasive device, STAN S31, could be used. This device allows the monitoring of the fHR and the performance of ST segment analysis with the use of the fetal scalp electrode attached to the fetal head or body [18,19,63].

### 4.3. Fetal ECG Processing Methods

Today, the non-invasive fECG has replaced the invasive approach, but it requires the use of advanced, sophisticated signal processing methods in order to extract the fECG signal in a suitable form.

The main interference sources are similar to those of most electrical signals (see Table 1): 50 Hz frequency interference, the baseline wander and the electromyogram interference. However, the main artifact for the non-invasive fECG is the maternal ECG (mECG). It is very difficult to precisely determine the fetal R peaks, because they overlap with the maternal ones. The amplitude of the mECG signal is several times higher than the amplitude of the fECG signal. The fact that the heart rates of mother and fetus are different needs to be taken into consideration. The fetal heart rate is normally in the range from 120 to 160 beats per minute (bpm) and the maternal heart rate (mHR) is in the range from 70 to 80 bpm [18,19,63].

Choosing a suitable signal processing method is very challenging. The signal processing methods can be classified as either adaptive or non-adaptive methods:

- **The adaptive methods** are based on a learning system. Such learning systems use previous experiences in order to improve the extraction accuracy of the fECG signals. Based on these experiences, the learning system automatically sets its own coefficients based on the external influences. The adaptive methods use the aECG signal as a primary input and the mECG signal as the reference signal. The most often used adaptive methods are the adaptive neuro-fuzzy inference system (ANFIS), the least mean squares (LMS) algorithm, and the recursive least squares (RLS) algorithm [18,63,133].
- **The non-adaptive methods** do not use any learning systems. These methods use only the aECG signals as an input in order to extract the fECG signals and are divided into the single channel signal source and the multichannel signal source methods. The most often used non-adaptive methods using a single channel signal source that includes the wavelet transform (WT), the template subtraction (TS), and the empirical mode decomposition (EMD). From the multichannel signal sources methods, the independent component analysis (ICA) and the principal component analysis (PCA) are the most often used approaches in studies and are presented in numerous publications [18,63,133].

The adaptive and non-adaptive methods have their advantages and disadvantages. For that reason, a great number of research teams try to use a combination of different methods to develop an appropriate *hybrid* method development. The ICA-based methods and some adaptive algorithms are very often used for the purpose of development of a hybrid method. Table 5 shows the summary of the most popular fECG processing methods [141,142,143,144,145].

#### 4.3.1. Adaptive Neuro-Fuzzy Inference System

The ANFIS is one of the most commonly used signal processing methods for the purpose of the fECG analysis, which was developed by Jang in 1993 [146], and is very often marked as a hybrid method, because it combines the Takagi–Sugeno fuzzy inference system (fuzzy logic) with the multi-layer forward neural network [146,147,148,149].

Swarnalatha and Prasad in 2010 [147] used the ANFIS for the mECG cancellation. They evaluated the accuracy of the ANFIS with the SNR, correlation coefficients and with the performance indices. They concluded that the ANFIS enabled the successful removal of the artifacts and the extraction of the desired fECG signal. Even better accuracy of the fECG extraction was achieved with the implementation of the WT for post-processing of the fECG signal.

Assaleh in 2006 [148] presented an efficient technique called ANFIS for the fECG extraction from a composite aECG recording. He introduced that the ANFIS operates on the two ECG signals recorded at the thoracic (mECG) and the abdominal (aECG) areas of the mother’s skin. The results showed that the fECG signal, after performing the ANFIS, was extracted by simply subtracting the aligned version of the mECG signal from the aECG signal.

Jothi and Prabha in 2014 [149] also tried to extract the fECG signal with the adaptive method (namely ANFIS) and then to improve the estimated fECG signal with an undecimated WT. They compared this approach with the DWT with the MSE between the denoised fECG signal and the original fECG signal. The experiment showed that the ANFIS with the undecimated WT provided significantly better accuracy than the DWT.

#### 4.3.2. Least Mean Squares

Least Mean Squares (LMS) is a basic representative of a stochastic gradient adaptive algorithms class based on the Wiener filtering theory, stochastic averaging and least squares. For the accurate fECG extraction it is very important to set the parameters appropriately. Constant μ is step size of the LMS filter and has a fundamental influence on both the speed and stability of the adaptive algorithm convergence. When the value of the applied step size is too small, then the calculation time of the LMS filter will be very long. The order of the applied filter also has a great influence and must be set appropriately [150,151,152,153].

Camps et al. in 2001 [150] used the classical version of the LMS and the normalized LMS (NLMS) for the fECG extraction. Results indicated that the LMS algorithm and its other versions are reliable methods for the fECG recovery. They concluded that this approach provides a robust method for the fECG extraction.

Sehamby and Singh in 2016 [151] implemented an LMS algorithm for non-invasive fECG signal processing. For evaluation purposes, the calculation of the SNR value was used. They concluded that the LMS algorithm is very simple and can be used for the real-time monitoring systems in order to improve the SNR and to reduce the noise.

#### 4.3.3. Recursive Least Squares

The Recursive Least Squares (RLS) algorithm is based on the recursive determination of the coefficient weights, the Kalman filtering theory, time averaging, and the LMS algorithm. This algorithm used previous error values, which improved the overall performance but was more computationally demanding and had problems with its stability. The fundamental influence on it was the forgetting factor (lies usually within the range from 0 to 1) and the applied filter order. Very often, the forgetting factor lies within the range from 0.95 to 1 [152,153,154].

Zeng et al. in 2008 [152] applied the RLS algorithm in order to eliminate the mECG signal and hence to extract the fECG signal from the composite aECG signal. The results showed that the RLS algorithm enables the speeding up of the convergence of the NLMS algorithm and allowed tracking of the non-stationary fECG signals in an adaptive manner. They concluded that the RLS algorithm could be used for real-time monitoring and offered more robustness than the LMS and the NLMS algorithms at the expense of increased algorithmic complexity.

Liu et al. in 2011 [153] also compared available RLS algorithms and with the LMS and the NLMS algorithms for the extraction of the fECG signals. Their results showed that the performance and accuracy of the RLS algorithms was more effective than the LMS and the NLMS algorithms in the adaptive manner, and it was found to converge faster than the LMS and the NLMS algorithms.

Kahankova et al. in 2017 [154] used the RLS algorithm based on the FIR adaptive filters for the estimation of the fECG signal from a mixture of the aECG signal and the measured mECG signal. They concluded that the optimal setting of the RLS algorithm filter length lays within the interval from 20 to 40, where the RLS algorithm showed the best possible accuracy while the computational time remained reasonable.

#### 4.3.4. Wavelet Transform

Karvounis et al. in 2004 [155] introduced a novel automated method for the fQRS complexes detection. Their method did not require a pre-processing step for noise filtering and was based on the CWT. They used actual fetal and maternal ECG signals recorded at different weeks of pregnancy for the validation of the extraction accuracy. The obtained results showed that the system worked well and almost all fetal beats were detected (the accuracy was 99.5%).

Hassanpour and Parsaei in 2006 [156] used the WT for the fECG signal processing. Their algorithm consisted of the two steps. The first one was the extraction of the fECG signal and was performed with a two-level WT. Then the fECG signal was low-pass filtered using the Savitzky–Golay smoothing filter to attenuate the effect of noise. Real and synthetic data were used for evaluation purposes and the obtained results showed that the proposed algorithm had a promising performance.

Deasi and Sankhe in 2012 [157] used the multiscale DWT for the real-time fECG feature extraction. They used signals from 35 different pregnant women for the evaluation purposes and the results showed that their system achieved more than 99.5% accuracy.

#### 4.3.5. Template Subtraction

This method is based on the repeatability of the mQRS complexes. First, the detection of all the mQRS complexes was performed and then the mean mQRS complex (template) was created. This template was then subtracted from all the mQRS complexes and only the fECG signal remained [158,159,160].

Agostinelli et al. in 2017 [158] used a template-based method called the segmented-beat modulation method for the aECG signals in order to achieve a signal-quality at least comparable to the direct fECG. In order to evaluate the accuracy of the proposed approach they used the calculation of both direct and indirect SNRs. They used the real data from abdominal measurements and the direct fECG (ADFECGDB) database [136,161], which contains five records from different women, which are 5 minutes long with a sampling frequency of 1 kHz and with 16-bit resolution. They concluded that the applied method is very accurate and can be used to obtain clean and a potentially clinically useful fECG signal comparable with the direct fECG signal.

Lipponen and Tarvainen in 2013 [159] introduced a new template-based method (augmented multi-lead principal component regression) for the mECG removal and the multichannel correlation based fHR detector. They used real data from the Physionet Challenge 2013 database [161,162], which consists of 175 four-channel abdominal fECG recordings with the durations of 1 min, 10 min, and 60 min, with the sampling frequency of 1 Hz and 12-bit resolution. Results showed that the applied template-based method succeeded in the removal of the mECG from the aECG signals with high accuracy.

Matonia et al. in 2006 [160] developed a new template-based method for the determination of the template maternal PQRST complex and its subtraction from all detected cycles in the aECG signal. Three recordings from different women were used for evaluation purposes. The obtained results showed that the proposed approach allowed the performance of the complete suppression of the mECG signal without affecting the fECG signal. They concluded that the quality of the estimated fECG signal could be improved with the use of a spatial filtering technique, because after performing the proposed approach noise caused by the abdominal muscle activity remained.

#### 4.3.6. Empirical Mode Decomposition

Ghosh and Poonia in 2015 [163] compared some of the EMD based techniques for fECG signal processing (reduction of the baseline wander). For comparison purposes they used the EMD, the ensemble EMD (EEMD) and the EMD based methods. They concluded that the approach based on the EMD method enabled the best reduction of the baseline wander.

Azbari et al. in 2017 [164] used the EMD as a part of the filtering system for fECG extraction from the composed aECG signal. They found out that the complete EEMD provided a better frequency resolution of modes and required a lower number of EMD iterations. The conclusion was that their approach allowed them to detect the R–R intervals with high accuracy and that the fECG signals could be estimated with high speed.

Kumar et al. in 2018 [91] provided an investigation of the effectiveness of the EMD method with the non-local mean technique (NLM) by using the value of the differential standard deviation for the purpose of the fECG estimation. For evaluation of performance, they used recordings from the MIT-BIH arrhythmia database, and they added white and color Gaussian noise to the chosen signals. Based on the calculation of the percent root mean square difference (PRD), the MSE, and the SNR improvement, their approach achieved better results than the following methods: EMD, NLM, WT, adaptive filtering and conventional filtering.

#### 4.3.7. Independent Component Analysis

Manorost et al. in 2017 [165] used the ICA algorithm to extract the fECG signals from the database [136,161]. After the pre-processing and the mECG extraction, the mECG signal from the raw aECG signals was reduced in order to estimate the fECG signal. For evaluation purposes, they used percentage determination of the average fHR between the reference and the extracted signal. They concluded that the ICA method achieved a 90.43% accuracy.

John et al. in 2017 [166] introduced an ambulatory fetal heart monitor connected to smartphones via Bluetooth. In order to estimate the fHR and the maternal heart rate (mHR) they used the ICA algorithm and then the Pan–Tompkins detector. For the evaluation of their solution’s accuracy, they used real data from the Physionet Challenge 2013 database [161,162]. They concluded that the combination of the ICA algorithm and the Pan–Tompkins detector provided a low rate of spurious detections and can be employed to successfully estimate the fHR and the mHR.

Kotas et al. in 2017 [167] used the ICA based algorithm, called the joint approximate diagonalization of eigenmatrices approach (JADE), for the fECG data estimation. This approach avoids redundancy, so the JADE algorithm basically reduces matrix and computational costs. They concluded that the JADE algorithm showed a fast and good performance.

#### 4.3.8. Principal Component Analysis

This method is one of the most frequently applied methods of multidimensional statistical analysis. The PCA works on the replacement principle of original correlated variables with new variables, which are not correlated. The main aim of using the PCA is to find components reducing the dimensionality of the input signal without loss of information. Basically, this method uses the decomposition of the input signal covariance matrix into numbers and vectors [133,168,169,170].

Bacharakis et al. in 1996 [168] compared the PCA algorithm with the higher-order singular value decomposition (SVD) and the higher-order eigen value decomposition (EVD). They used 8 real recordings for the carried out experiment and the results showed that the PCA algorithm is not as accurate as the other methods they compared it with.

Petrolis et al. in 2013 [169] used the PCA algorithm for the elimination of the mECG from the aECG signals. For the experiment, they used real data from the Physionet Challenge 2013 database [161,162]. Their results showed that the applied PCA algorithm provided promising accuracy in the fECG extraction process.

Raj et al. in 2015 [170] compared the PCA with the ICA algorithm for the purpose of the estimation of the fECG. As the evaluation parameters, they have chosen the calculation of the correlation coefficient and the bpm determination. Real data from the database [136,161] and from the Physionet Challenge 2013 database [161,162] were used in order to conduct the experiments. They concluded that the both blind source separation (BSS) algorithms have practically the same accuracy during the fECG extraction.

#### 4.3.9. Hybrid Methods

Hybrid methods are created with a combination of at least 2 methods for the purpose of using the advantages from each of the applied methods. These methods have great potential to be used in real-time monitoring systems applied for checking fetal well-being. The ICA algorithm is one of the most frequently used methods for the creation of the hybrid method. The EMD, the WT and some adaptive filters are also often used as a part of the hybrid system.

Liu and Luan 2015 [142] proposed a novel integrated adaptive algorithm based on the ICA, the EEMD, and the wavelet shrinkage (WS) denoising (hybrid method called ICA-EEMD-WS). For the experiment’s purpose, they compared this approach with the Butterworth filter, the WS, and the hybrid method EEMD-WS. They applied both generated data and real data from the database [136,161]. The comparison was made with a calculation of the SNR, the MSE and the coefficient correlation. The proposed hybrid method ICA-EEMD-RLS achieved the best results from all the tested methods and the authors of that work concluded that if the EEMD method was faster then this approach could be used for the real-time monitoring of the fHR and the mHR.

Alvarez et al. in 2015 [171] used the PCA and the ICA algorithms and combined them in order to develop a hybrid method for fECG extraction. They compared this approach with the JADE algorithm. For the purpose of this experiment, they used a semi-synthetic database compound of the 26 aECG recordings. Results were obtained with a calculation of the SNR and they concluded that this method provided better accuracy than the JADE algorithm.

Billeci and Varanini et al. in 2017 [172] proposed a novel fECG algorithm based on the ICA and the quality index optimization (QIO), which made a new hybrid method (the ICAQIO-based method). They compared this hybrid method with the ICA and the QIO by way of synthetic data from the fECG synthetic database (FECGSYNDB) and the real data from the Physionet Challenge 2013 database [161,162]. The proposed hybrid ICAQIO-based method outperformed the other two algorithms on the generated data, but on the real data the hybrid ICAQIO-based method achieved practically the same accuracy as the QIO. They came to the conclusion that the hybrid ICAQIO-based method could be effectively used for fECG extraction, when the uterine contractions are present and the maternal and the fetal ectopic beats occur.

Panigrahy and Sahu et al. in 2017 [173] investigated the use of the combination the ANFIS method with the extended Kalman filter for the purpose of the fECG estimation from one aECG signal recorded from the electrodes placed on the maternal abdomen. The extended Kalman filter was used for the estimation of the mECG signal and the ANFIS algorithm was then applied for the identification of a nonlinear relationship between the estimated mECG and the actual mECG contained in the raw aECG signal. The fECG signal was extracted with a subtraction of the aligned version of the estimated mECG signal from the aECG signal. They evaluated this approach using both generated and clinically-collected data and concluded that this hybrid method is accurate for the case of singleton pregnancy, unless the aECG signal is strongly contaminated with the environment-based noise.

## 5. Discussion

This paper is a Part 1 paper and it focuses on the classification of various bioelectrical signals (their definition) and in particular on cardiac signals, providing insight into their specific characteristics, measurement standards, and their applicability in clinical practice. The paper in particular aims to present a thorough summary of various interferences and artifacts that co-occur with these signals and adversely affect them. This work also covers a brief introduction of the methods applied for the purpose of the elimination of interferences. Undoubtedly, the interference present in the analyzed signal can prevent the proper analysis of the signal, so its suppression is essential in many cases. Based on a thorough literature study, the signal processing methods can be divided into several groups:

1. Classical digital filtering and adaptive filtering;
2. Advanced signal processing methods, such as the WT, the ICA (Independent Component Analysis), the PCA (Principal Component Analysis), the EMD and other new methods;
3. Various modifications and combinations of the previously listed methods.

The choice of methods providing the best performance depends on the type of signal (due to the different frequency ranges, amplitude spectrum, etc.) and the type of co-occurring interference (narrow-band, broadband, impulse noise, overlapping in frequency, etc.).

Standard or classical digital filtering can be used when the interference and the desired biological signals have different frequency ranges. On the contrary, using the frequency-domain based filtering methods enables significant distortion of the signals’ frequency content and, thus, the obtaining of the appropriate diagnostic information. This technique is currently widespread in clinical practice as it provides a basic distinction of the signals’ frequency ranges (e.g., filtering of high-frequency noise and baseline wandering). The adaptive noise cancelling technique is a better solution when overlapping the frequency spectra of the interference and the signal, providing accurate filtering results. However, the need for a reference signal means a slight complication during recording, when the special sensor/s are necessary in order to obtain only the interference, which has to be eliminated. This can lead to decreased patient comfort and a higher cost of the whole examination procedure.

## Figures and Tables

**Table 1:** Classification of bioelectrical signals.

Bioelectrical Signal	Signal Origin	Frequency Range (Hz)	Typical Amplitude (mV)	Measurement Method
ECG (Electrocardiogram) [15,16,17]	Action potentials of heart muscle cells	0.05–250	0.01–5	surface
fECG (Fetal ECG) [18,19]	Fetal heart activity	0.05–250	0.01–0.02	surface
VCG (Vectorcardiogram) [20,21]	Action potentials of heart muscle cells			surface
EEG (Electroencephalogram) [15,22,23]	Brain neurons activity	0.1–80	0.005–0.3 0.005–10	surface intracortical
EP (Evoked Potentials) [15,24,25]	Brain activity in reaction on external stimuli	30–3000	0.0001–0.02	surface
ECoG (Electrocorticogram) [26,27]	Signal generated by cerebral cortex	0.1–100	0.005–10	surface
ENG (Electroneurogram) [28,29]	Action potentials of peripheral nerves	0.01–1000	0.005–10	interstitial
EMG (Electromyogram) [15,30,31]	Action potentials of muscle fibers	0.01–10,000	0.1–10 0.05–0.3	surface
EGG (Electrogastrogram) [15,32,33]	Gastric muscles activity	0.02–0.15	0.01–0.5 0.1–10	surface intragastric
EOG (Electrooculogram) [34,35]	Stiff muscles activity	0.5–15	0.05–3.5	surface
ERG (Electroretinogram) [36,37]	Eye retina activity	0.2–50	0.005–1	surface
EHG (Electrohysterogram) [38,39,40]	Uterus activity during contractions	0.1–3	0.1–5 0.1–1	surface intrauterine

**Table 3:** Summary of the ECG signal processing methods.

Method	Overall Performance	SNR Improvement	Computational Cost	Real-Time	Implementation Complexity
DF	Low	Medium	Low	Yes	Simple
DWT	Low	Medium	Medium	Yes	Medium
ANC	High	Medium	Medium	Yes	Medium
EMD	Medium	Medium	High	No	Medium
Neural Networks	High	Medium	Medium	Yes	Complex
Clustering	High	High	High	No	Complex
Hybrid Methods	High	High	High	Yes	Complex

**Table 4:** Summary of the VCG signal processing methods.

Method	Overall Performance	SNR Improvement	Computational Cost	Real-Time	Implementation Complexity
DF	Low	Medium	Low	Yes	Simple
DWT	Low	High	Medium	Yes	Medium
MA	Low	Medium	Low	Yes	Simple
KF	Low	Medium	Medium	No	Medium
SG	Medium	Medium	Low	Yes	Medium
PCR	Medium	Medium	Medium	Yes	Medium

**Table 5:** Summary of the fECG signal processing methods.

Method	Overall Performance	SNR Improvement	Computational Cost	Real-Time	Implementation Complexity
ANFIS	Medium	Medium	High	No	Complex
LMS	Medium	Medium	Medium	Yes	Simple
RLS	Medium	Medium	Medium	No	Simple
DWT	Medium	Medium	Low	Yes	Medium
TS	Medium	Low	Low	Yes	Simple
EMD	Medium	Medium	High	No	Medium
ICA	Medium	Medium	Medium	No	Medium
PCA	Low	Medium	Low	Yes	Simple
Hybrid methods	High	High	High	Yes	Complex
